# Supplementary material for: Integrated extracellular microRNA profiling for ovarian cancer screening
Source: Nat Commun. 2018 Oct 17;9:4319. doi: 10.1038/s41467-018-06434-4 (PMC6192980; doi:10.1038/s41467-018-06434-4)
Supplement: Supplementary file 1 — Supplementary Information [file 41467_2018_6434_MOESM1_ESM.pdf]

Supplementary Information

Integrated extracellular microRNA profiling for ovarian cancer screening

Yokoi et al.

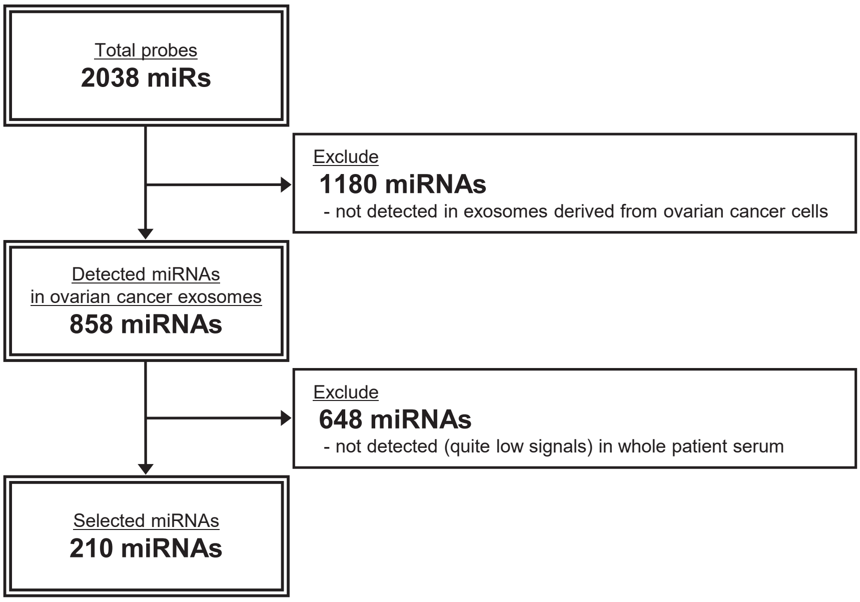

Supplementary Figure 1. Flow diagram for selection of miRNAs. (Patient-based) According to the results of microarray analysis for 442 patients with ovarian tumors, 363 miRNAs were selected as meaningful candidates. According to analyses for exosomes derived from 12 ovarian cancer cell lines, 858 miRNAs were detected. As shown in Venn diagram, 210 miRNAs overlapped.

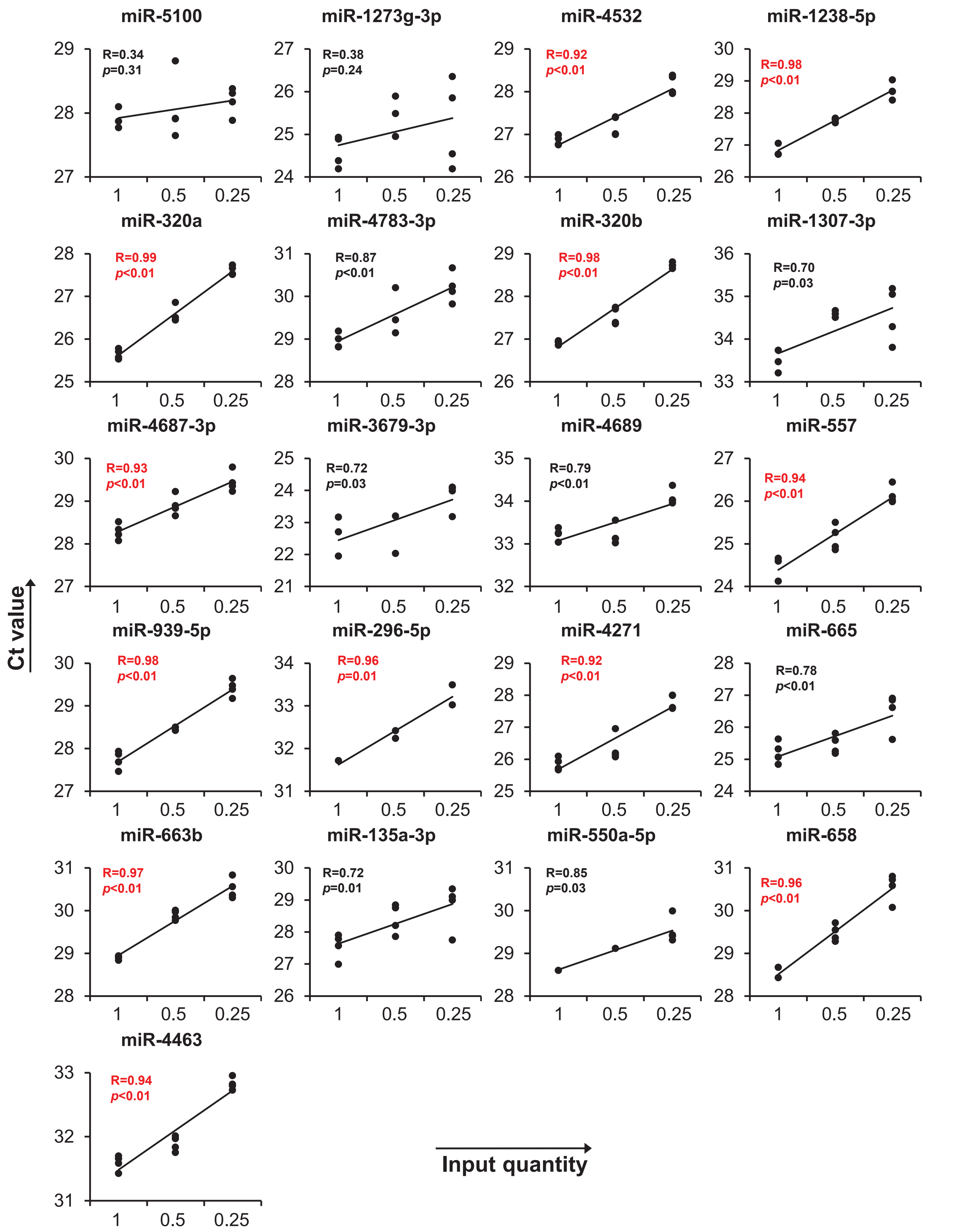

Supplementary Figure 2. Quantitative performance of pivot miRNAs by qRT-PCR To check the quantitativity of the analysis 3 different concentration (relative concentration 1, 0.5 and 0.25) of samples were analyzed. Among total 25 miRNAs which were selected as pivot miRNAs in model 1-3, four miRNAs were excluded due to non-reaction by qRT-PCR (miR-1223-5p, miR-4787-3p, miR-4739) and non-exist primer (miR-4294). The R and *p* values were calculated using Pearson's correlation analysis.

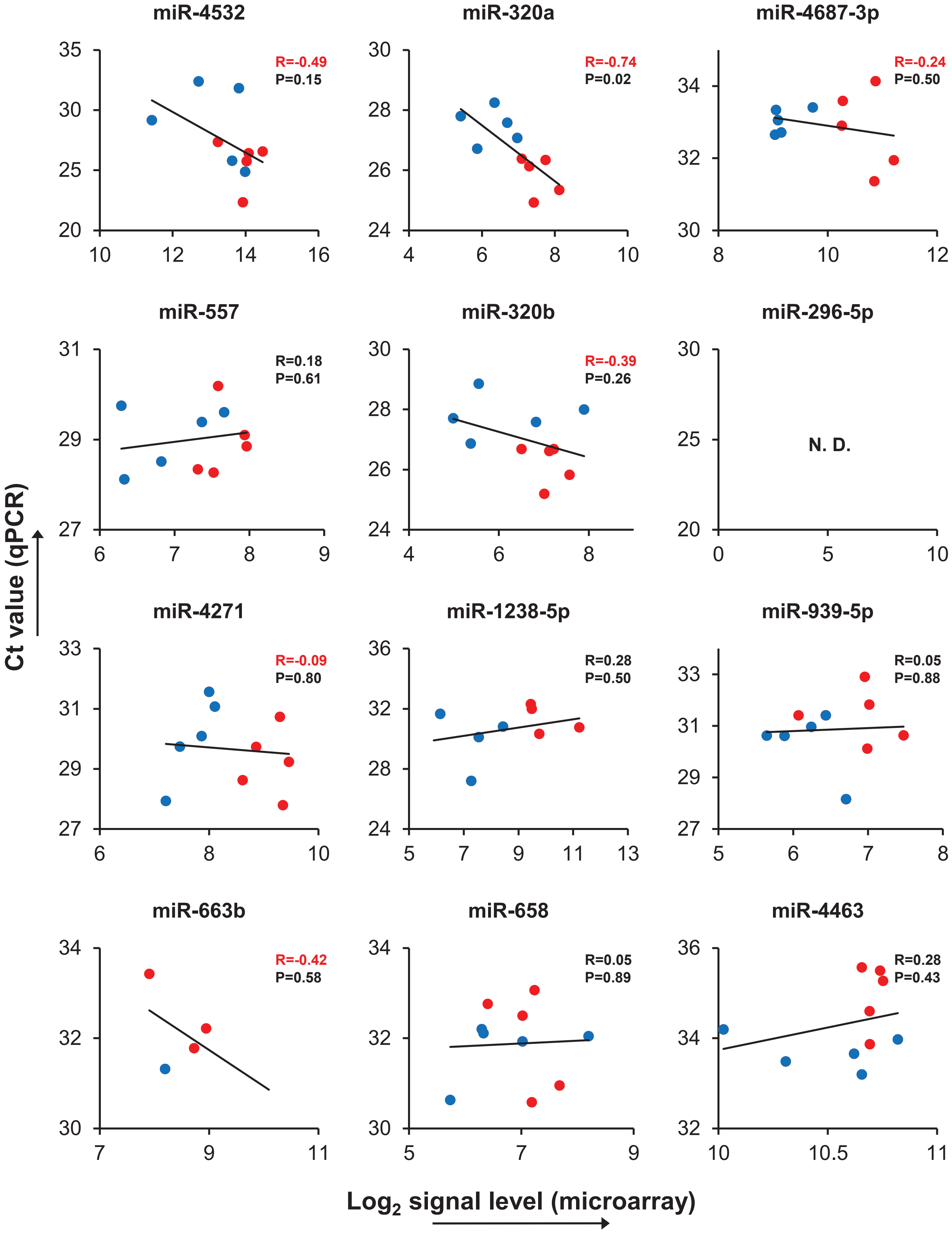

Supplementary Figure 3. Corelation of miRNA expression between microarray and qRT-PCR
The expression of each miRNAs measured by qRT-PCR and microarray was plotted on the histograms. The R and *p* values were calculated using Pearson's correlation analysis.

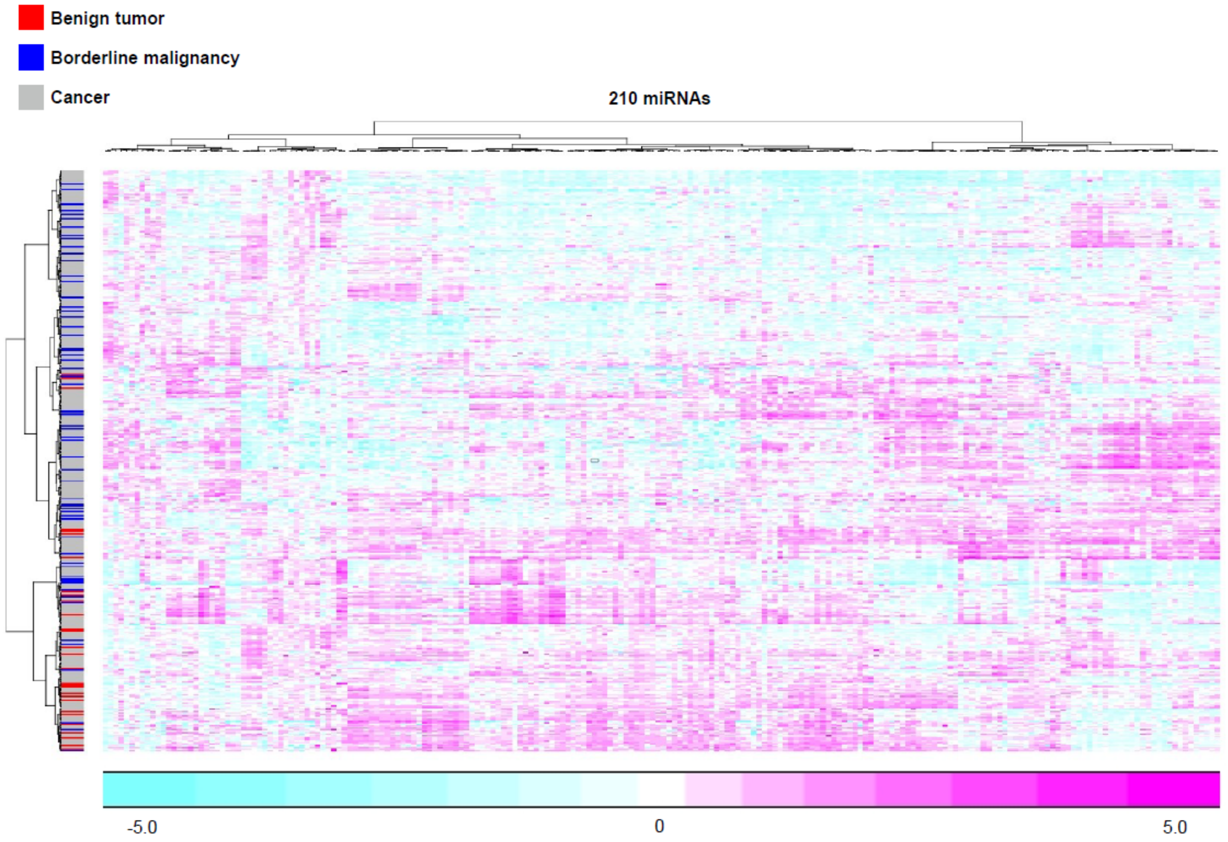

Supplementary Figure 4. Heat map for serum miRNA expression of patients with benign tumors, borderline tumors or cancer For gene expression analysis, a one-way analysis of variance was performed to identify differentially expressed genes ($P < 0.05$), and 108 miRNAs were selected from 210 miRNAs. Unsupervised clustering and heat map generation were performed with sorted datasets using Pearson's correlation in Ward's method with selected probe sets and Partek Genomics Suite 6.6. N = Cancer; 333, Borderline malignancy; 66 and Benign tumor; 29.

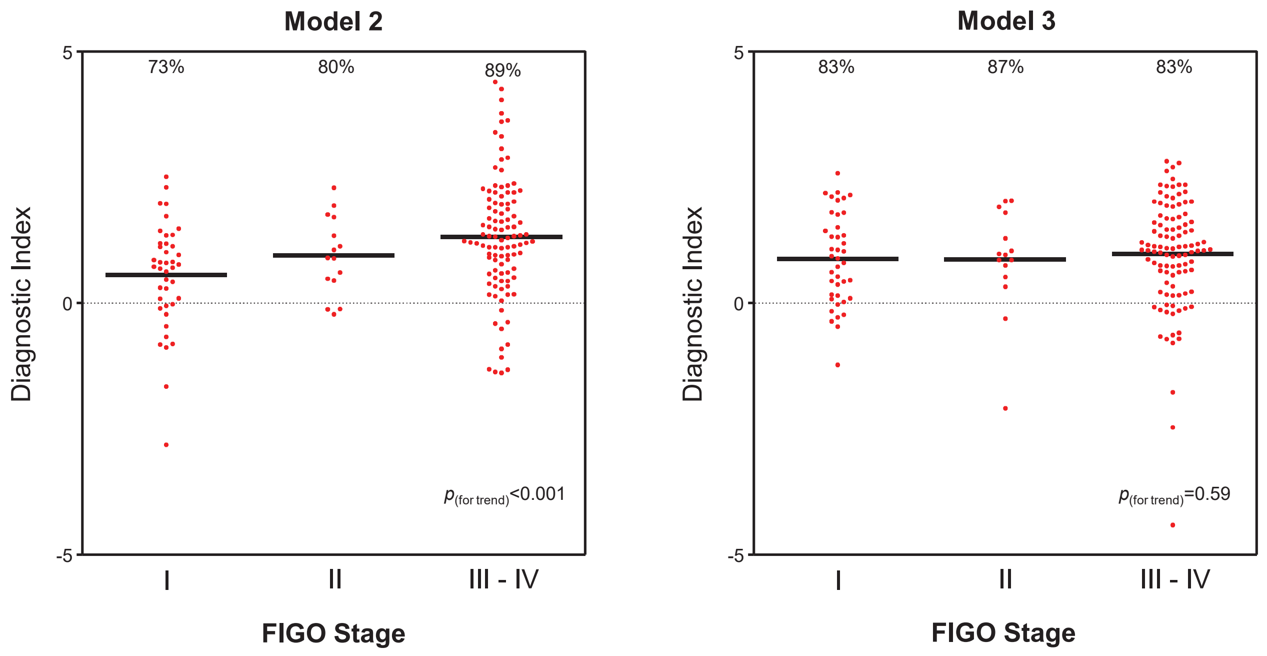

Supplementary Figure 5. Diagnostic performance of models for FIGO stages The diagnostic index for each FIGO stage using prediction model 2 and 3. N = stage I; 82, stage II; 33 and stage III-IV; 218. The *p* values were calculated using Pearson's correlation analysis.

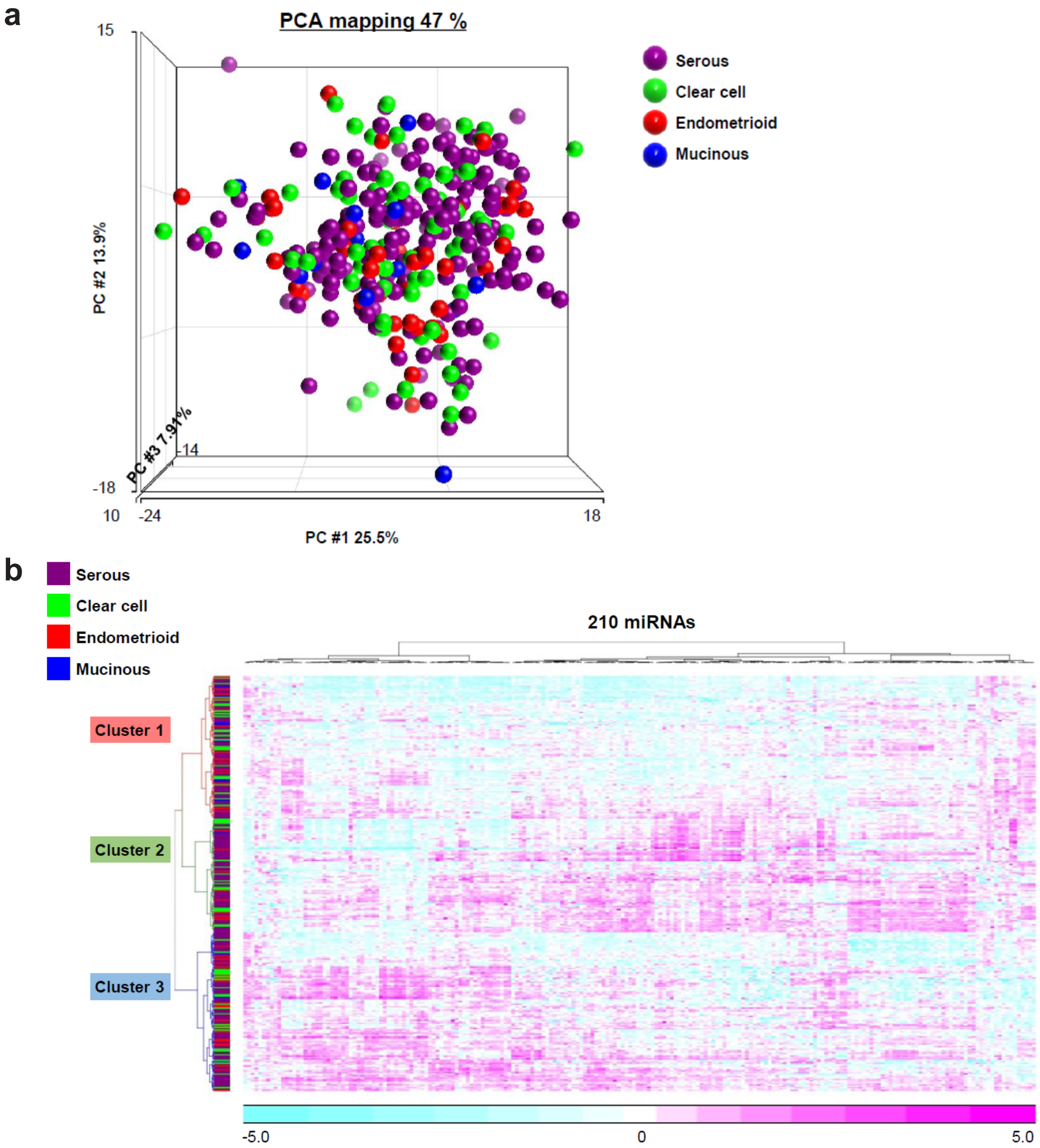

Supplementary Figure 6. Serum miRNA expression of each histopathological subtype a. PCA mapping (a) and Heat map (b) for serum miRNA expression of each histopathological subtype. For gene expression analysis, a one-way analysis of variance was performed to identify differentially expressed genes ($P < 0.05$), and 108 miRNAs were selected from 210 miRNAs. Unsupervised clustering and heat map generation were performed with sorted datasets using Pearson's correlation in Ward's method with selected probe sets and Partek Genomics Suite 6.6. N = serous; 182, clear-cell; 64, endometrioid; 43 and Mucinous; 14.

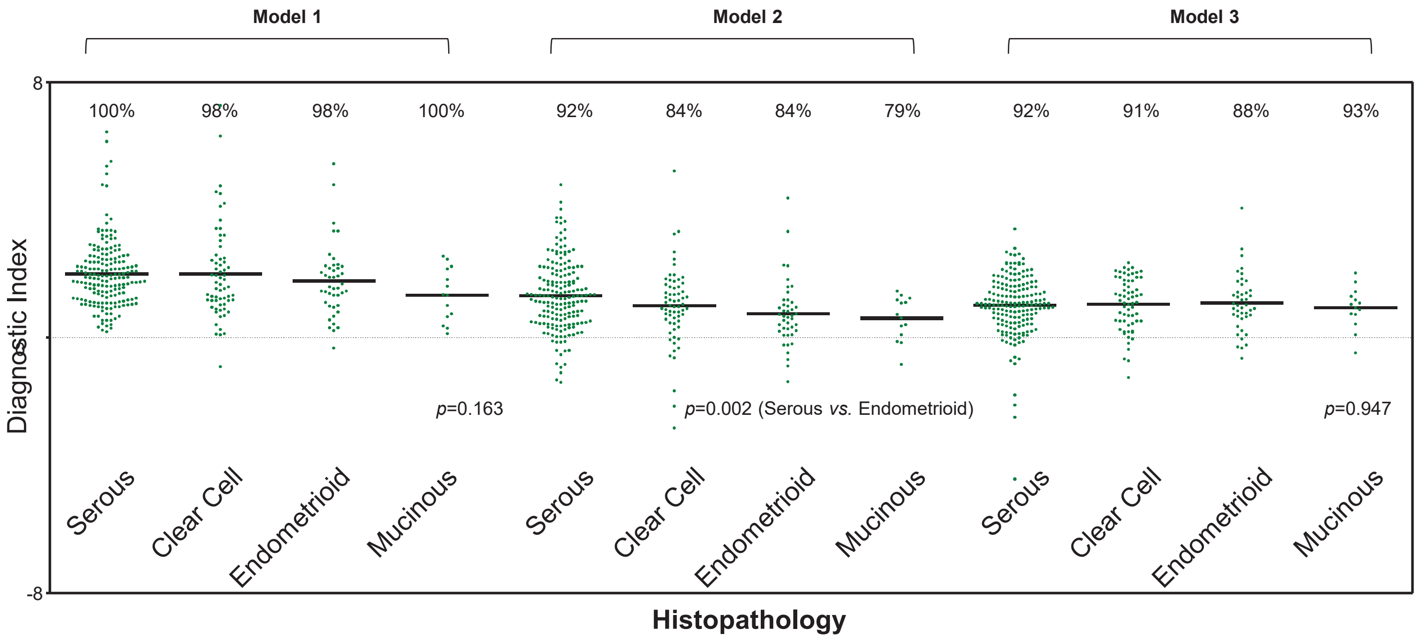

Supplementary Figure 7. Diagnostic performance of the models for histopathological subtypes

The diagnostic index for each histopathological subtype using a prediction model 1, 2 and 3. N = serous; 182, clear-cell; 64, endometrioid; 43 and Mucinous; 14. The *p* value was calculated using one-way ANOVA (analysis of variance) and Tukey's post hoc analysis.

Supplementary Table 1. Ovarian cancer cell lines

Cell lines	Subtypes	Source	Cat. no.
SKOV3	NS	ATCC	HTB-77
NIH-OVCAR3	NS	ATCC	HTB-161
CAOV3	NS	ATCC	HTB-75
ES-2	Clear cell	ATCC	CRL-1978
OV-90	Serous	ATCC	CRL-11732
A2780	NS	ECACC	93112519
OAW42	Serous	ECACC	85073102
COV362	Endometrioid	ECACC	7071910
MCAS	Mucinous	JRBC	JCRB0240
RMG-I	Clear cell	JRBC	IFO50315
RMUG-S	Mucinous	JRBC	IFO50320
KURAMOCHI	NS	JRBC	JCRB0098

Supplementary Table 2. Candidates of pivot miRNAs for model 1

	Cross-validated accuracy	AUC
miR-5100	0.94	0.95
miR-1273g-3p	0.94	0.94
miR-4532	0.94	0.97
miR-1238-5p	0.94	0.94
miR-1233-5p	0.94	0.97
miR-320a	0.93	0.96
miR-4787-3p	0.93	0.94
miR-4783-3p	0.93	0.96
miR-320b	0.93	0.95
miR-1307-3p	0.93	0.94

Supplementary Table 3. Candidates of model 1

Number of miRNAs	Model candidates	Sensitivity	Specificity	Accuracy	AUC
1	(1.32939)*miR-4532-16.479	0.956	0.928	0.931	0.974
1	(1.079)*miR-320a-6.192	0.963	0.897	0.904	0.962
1	(0.67932)*miR-320b-3.657	0.931	0.933	0.932	0.954
2	(0.426799)*miR-320b+(1.43959)*miR-3663-3p-19.783	0.975	0.941	0.944	0.984
2	(0.381037)*miR-320b+(0.952211)*miR-1273g-3p-9.231	0.944	0.921	0.923	0.972
2	(0.468814)*miR-320b+(1.61028)*miR-296-5p-14.712	0.981	0.927	0.932	0.985
2	(0.994113)*miR-320a+(1.03855)*miR-3195-14.759	0.956	0.956	0.956	0.979
2	(0.594309)*miR-320b+(1.20287)*miR-3195-13.145	0.931	0.938	0.938	0.968
2	(0.498724)*miR-320b+(0.846838)*miR-658-7.6457	0.963	0.922	0.927	0.972
2	(1.28333)*miR-4532+(0.539073)*miR-1292-3p-18.680	0.981	0.920	0.927	0.985
2	(0.951583)*miR-320a+(0.915273)*miR-718-11.930	0.956	0.934	0.936	0.975
2	(0.56336)*miR-320b+(1.16408)*miR-718-11.033	0.919	0.946	0.944	0.968
2	(0.927269)*miR-320a+(0.981676)*miR-665-12.529	0.944	0.932	0.933	0.976
3	(0.246463)*miR-320b+(1.01641)*miR-665+(-0.818026)*miR-3184-5p-3.208	0.994	0.954	0.958	0.991
3	(0.382431)*miR-320b+(1.17589)*miR-665+(-1.77092)*miR-4741+5.816	0.950	0.963	0.962	0.982
3	(0.290673)*miR-320b+(1.01409)*miR-1273g-3p+(-0.720778)*miR-760-4.055	0.950	0.962	0.960	0.987
3	(0.52068)*miR-320a+(0.99356)*miR-665+(-0.67811)*miR-3184-5p-5.602	1.000	0.939	0.945	0.991
3	(0.403865)*miR-320b+(1.21136)*miR-1273g-3p+(-0.833395)*miR-3135b-5.128	0.944	0.962	0.960	0.982
3	(0.354245)*miR-320b+(0.978417)*miR-1273g-3p+(-0.670256)*miR-4667-5p-5.532	0.950	0.964	0.962	0.987
3	(0.757734)*miR-320a+(1.08713)*miR-665+(-1.50847)*miR-4741+1.524	0.969	0.951	0.953	0.991
3	(0.902745)*miR-320a+(0.904003)*miR-3195+(-0.654885)*miR-711-7.777	0.975	0.951	0.954	0.986
3	(0.458674)*miR-320b+(1.35437)*miR-296-5p+(0.537255)*miR-718-16.530	0.975	0.942	0.945	0.988
3	(0.324564)*miR-320b+(1.28215)*miR-296-5p+(0.61384)*miR-4787-3p-15.482	0.981	0.952	0.955	0.990
4	(0.301051)*miR-320b+(0.988794)*miR-665+(-0.847117)*miR-3184-5p+(-0.145975)*miR-6717-5p-2.511	0.981	0.979	0.979	0.993
4	(0.737169)*miR-320a+(0.935278)*miR-665+(-0.679304)*miR-3184-5p+(-0.278991)*miR-6717-5p-5.487	0.981	0.980	0.981	0.994
4	(0.242103)*miR-320b+(1.07486)*miR-665+(-0.781358)*miR-3184-5p+(-0.884166)*miR-6089+7.741	0.988	0.963	0.966	0.991
4	(0.254379)*miR-320b+(1.04465)*miR-665+(-0.817563)*miR-3184-5p+(-0.111791)*miR-4430-3.003	0.981	0.970	0.971	0.992
4	(0.243709)*miR-320b+(1.10585)*miR-665+(-0.814265)*miR-3184-5p+(-0.21653)*miR-486-3p-2.139	0.994	0.959	0.963	0.991
4	(0.25685)*miR-320b+(1.06795)*miR-665+(-0.844351)*miR-3184-5p+(-0.19947)*miR-4298-2.504	0.975	0.974	0.974	0.992
4	(0.259106)*miR-320b+(1.00845)*miR-665+(-0.828538)*miR-3184-5p+(-0.140621)*miR-365a-5p-2.302	0.994	0.963	0.966	0.992
4	(0.258766)*miR-320b+(1.03642)*miR-665+(-0.758155)*miR-3184-5p+(-0.285368)*miR-4667-5p-2.085	0.994	0.957	0.960	0.992
4	(0.251889)*miR-320b+(1.06945)*miR-665+(-0.816572)*miR-3184-5p+(-0.256273)*miR-4688-1.927	0.988	0.966	0.968	0.992
4	(0.254288)*miR-320b+(1.04641)*miR-665+(-0.784831)*miR-3184-5p+(-0.101129)*miR-4507-2.980	1.000	0.946	0.951	0.991

5	(0.731913)*miR-320a+(0.908322)*miR-665+(-0.631772)*miR-3184-5p+(-0.256159)*miR-6717-5p+(-0.479172)*miR-6125-0.060	0.988	0.988	0.988	0.994
5	(0.702294)*miR-320a+(1.02996)*miR-665+(-0.692446)*miR-3184-5p+(-0.30643)*miR-6717-5p+(-0.344592)*miR-4459-2.619	0.981	0.985	0.984	0.994
5	(0.645869)*miR-320a+(0.803688)*miR-665+(-0.638787)*miR-3184-5p+(-0.271653)*miR-6717-5p+(0.498912)*miR-3663-3p-10.449	0.981	0.986	0.985	0.995
5	(0.248451)*miR-320b+(1.00141)*miR-665+(-0.739886)*miR-3184-5p+(-0.259256)*miR-6717-5p+(0.374884)*miR-4454-5.920	0.994	0.974	0.976	0.994
5	(0.736232)*miR-320a+(0.94844)*miR-665+(-0.669113)*miR-3184-5p+(-0.277674)*miR-6717-5p+(-0.554135)*miR-6085+0.115	0.988	0.981	0.982	0.994
5	(0.669529)*miR-320a+(0.807274)*miR-665+(-0.630728)*miR-3184-5p+(-0.236674)*miR-6717-5p+(0.428068)*miR-296-5p-8.086	0.981	0.983	0.983	0.995
5	(0.251539)*miR-320b+(0.980088)*miR-665+(-0.801751)*miR-3184-5p+(-0.241971)*miR-6717-5p+(0.0866708)*miR-1246-2.529	0.975	0.985	0.984	0.993
5	(0.299135)*miR-320b+(0.957389)*miR-665+(-0.861115)*miR-3184-5p+(-0.144022)*miR-6717-5p+(0.356873)*miR-1237-5p-6.657	0.981	0.977	0.977	0.993
5	(0.297745)*miR-320b+(0.947257)*miR-665+(-0.813951)*miR-3184-5p+(-0.182836)*miR-6717-5p+(0.0785589)*miR-4710-2.800	0.981	0.978	0.979	0.993
5	(0.696094)*miR-320a+(0.911961)*miR-665+(-0.586036)*miR-3184-5p+(-0.269428)*miR-6717-5p+(-0.182797)*miR-575-4.673	0.981	0.983	0.983	0.994
6	(0.23422)*miR-320b+(0.754343)*miR-665+(-0.702193)*miR-3184-5p+(-0.253298)*miR-6717-5p+(0.110379)*miR-1246+(0.614481)*miR-6515-3p-5.856	0.988	0.986	0.986	0.995
6	(0.241972)*miR-320b+(0.634398)*miR-665+(-0.7577)*miR-3184-5p+(-0.238138)*miR-6717-5p+(0.641957)*miR-1238-5p+(0.973031)*miR-4466-16.659	0.988	0.988	0.988	0.995
6	(0.684291)*miR-320a+(0.846601)*miR-665+(-0.763983)*miR-3184-5p+(-0.298476)*miR-6717-5p+(-0.423458)*miR-4459+(0.673605)*miR-557-4.637	0.988	0.988	0.988	0.996
6	(0.251073)*miR-320b+(1.01901)*miR-665+(-0.739596)*miR-3184-5p+(-0.251054)*miR-6717-5p+(0.370586)*miR-4454+(-0.0644493)*miR-4430-5.709	0.994	0.975	0.977	0.994
6	(0.718882)*miR-320a+(0.831044)*miR-665+(-0.645722)*miR-3184-5p+(-0.253269)*miR-6717-5p+(-0.586076)*miR-6125+(0.44757)*miR-762-3.708	0.994	0.983	0.984	0.995
6	(0.694888)*miR-320a+(0.852167)*miR-665+(-0.587791)*miR-3184-5p+(-0.254159)*miR-6717-5p+(-0.875348)*miR-6125+(0.598555)*miR-5787-2.887	0.994	0.986	0.986	0.995
6	(0.712445)*miR-320a+(0.918645)*miR-665+(-0.594721)*miR-3184-5p+(-0.25036)*miR-6717-5p+(-0.397001)*miR-6125+(-0.283554)*miR-711+1.248	0.994	0.980	0.982	0.995
6	(0.702147)*miR-320a+(0.757069)*miR-665+(-0.662784)*miR-3184-5p+(-0.231023)*miR-6717-5p+(-0.777809)*miR-6125+(0.476174)*miR-4634+0.958	1.000	0.977	0.979	0.995
6	(0.679821)*miR-320a+(1.02117)*miR-665+(-0.74037)*miR-3184-5p+(-0.299035)*miR-6717-5p+(-0.894412)*miR-4459+(0.57792)*miR-6076-1.227	0.994	0.985	0.986	0.995
6	(0.243623)*miR-320b+(1.01919)*miR-665+(-0.715364)*miR-3184-5p+(-0.239808)*miR-6717-5p+(0.374666)*miR-4454+(-0.201747)*miR-4486-4.855	0.994	0.975	0.977	0.994
7	(0.604089)*miR-320a+(0.494465)*miR-665+(-0.647798)*miR-3184-5p+(-0.267378)*miR-6717-5p+(-0.580969)*miR-6125+(0.601944)*miR-4634+(0.499184)*miR-1238-5p-3.564	1.000	0.991	0.992	0.997
7	(0.641386)*miR-320a+(0.839155)*miR-665+(-0.756689)*miR-3184-5p+(-0.253311)*miR-6717-5p+(-0.804783)*miR-4459+(0.576048)*miR-6076+(0.643392)*miR-3195-5.817	1.000	0.978	0.981	0.996

7	(0.243083)*miR-320b+(0.532397)*miR-665+(-0.812257)*miR-3184-5p+(-0.182683)*miR-6717-5p+(0.544186)*miR-1238-5p+(0.927498)*miR-4634+(-1.27649)*miR-6089+5.829	0.994	0.991	0.991	0.997
7	(0.679002)*miR-320a+(0.817854)*miR-665+(-0.778771)*miR-3184-5p+(-0.295321)*miR-6717-5p+(-0.422066)*miR-4459+(0.679587)*miR-557+(0.314929)*miR-1237-5p-8.373	0.988	0.989	0.989	0.997
7	(0.666239)*miR-320a+(0.788515)*miR-665+(-0.783357)*miR-3184-5p+(-0.304757)*miR-6717-5p+(-0.370532)*miR-4459+(0.768939)*miR-557+(0.546024)*miR-4466-11.982	0.994	0.988	0.989	0.996
7	(0.715035)*miR-320a+(0.879043)*miR-665+(-0.832069)*miR-3184-5p+(-0.289454)*miR-6717-5p+(-0.554971)*miR-4459+(0.729659)*miR-557+(0.310956)*miR-4530-6.817	0.988	0.989	0.989	0.996
7	(0.621691)*miR-320a+(0.959971)*miR-665+(-0.700671)*miR-3184-5p+(-0.328795)*miR-6717-5p+(-1.22465)*miR-4459+(0.879764)*miR-6076+(1.40838)*miR-4417-11.316	0.994	0.985	0.986	0.996
7	(0.676194)*miR-320a+(0.995769)*miR-665+(-0.695414)*miR-3184-5p+(-0.27793)*miR-6717-5p+(-0.876938)*miR-4459+(0.563702)*miR-6076+(-0.442323)*miR-6125+3.758	1.000	0.987	0.988	0.995
7	(0.653198)*miR-320a+(0.805666)*miR-665+(-0.58417)*miR-3184-5p+(-0.330135)*miR-6717-5p+(-0.896505)*miR-6125+(0.0898269)*miR-1246+(0.771239)*miR-1237-5p-3.758	1.000	0.988	0.990	0.997
7	(0.215044)*miR-320b+(0.632211)*miR-665+(-0.665601)*miR-3184-5p+(-0.226338)*miR-6717-5p+(0.671172)*miR-1238-5p+(1.00129)*miR-4466+(-0.526589)*miR-711-13.530	0.994	0.988	0.988	0.996
8	(0.629036)*miR-320a+(0.566352)*miR-665+(-0.697022)*miR-3184-5p+(-0.2689)*miR-6717-5p+(-0.589815)*miR-6125+(0.654499)*miR-4634+(0.595701)*miR-1238-5p+(-0.308463)*miR-211-3p-2.728	0.994	0.991	0.992	0.996
8	(0.634735)*miR-320a+(0.485232)*miR-665+(-0.641159)*miR-3184-5p+(-0.244052)*miR-6717-5p+(-0.512635)*miR-6125+(0.501975)*miR-4634+(0.561538)*miR-1238-5p+(-0.293567)*miR-365a-5p-2.439	1.000	0.991	0.992	0.997
8	(0.546075)*miR-320a+(0.515362)*miR-665+(-0.61984)*miR-3184-5p+(-0.302397)*miR-6717-5p+(-0.63267)*miR-6125+(0.572278)*miR-4634+(0.487643)*miR-1238-5p+(0.0611839)*miR-191-5p-2.663	1.000	0.991	0.992	0.997
8	(0.603311)*miR-320a+(0.539485)*miR-665+(-0.653821)*miR-3184-5p+(-0.274843)*miR-6717-5p+(-0.25304)*miR-6125+(0.453983)*miR-4634+(0.658065)*miR-1238-5p+(-0.687444)*miR-4749-5p-1.705	1.000	0.989	0.990	0.997
8	(0.227965)*miR-320b+(0.555905)*miR-665+(-0.749076)*miR-3184-5p+(-0.175377)*miR-6717-5p+(0.557762)*miR-1238-5p+(0.831461)*miR-4634+(-1.1874)*miR-6089+(-0.325633)*miR-711+7.459	0.994	0.991	0.992	0.997
8	(0.639098)*miR-320a+(0.432975)*miR-665+(-0.687927)*miR-3184-5p+(-0.244875)*miR-6717-5p+(-0.537779)*miR-6125+(0.685979)*miR-4634+(0.547071)*miR-1238-5p+(-0.120346)*miR-614-3.567	1.000	0.988	0.990	0.997
8	(0.510687)*miR-320a+(0.46598)*miR-665+(-0.641054)*miR-3184-5p+(-0.285525)*miR-6717-5p+(-0.581748)*miR-6125+(0.612845)*miR-4634+(0.490186)*miR-1238-5p+(0.114443)*miR-320b-3.243	1.000	0.988	0.990	0.997

8	(0.618463)*miR-320a+(0.470957)*miR-665+(-0.676722)*miR-3184-5p+(-0.24455)*miR-6717-5p+(-0.520619)*miR-6125+(0.664936)*miR-4634+(0.575946)*miR-1238-5p+(-0.0948278)*miR-2467-3p-4.703	0.994	0.993	0.993	0.997
8	(0.604978)*miR-320a+(0.574698)*miR-665+(-0.632912)*miR-3184-5p+(-0.272977)*miR-6717-5p+(-0.554652)*miR-6125+(0.542533)*miR-4634+(0.558686)*miR-1238-5p+(-0.449152)*miR-4739+0.779	1.000	0.991	0.992	0.997
8	(0.647787)*miR-320a+(0.511347)*miR-665+(-0.641861)*miR-3184-5p+(-0.220977)*miR-6717-5p+(-0.614861)*miR-6125+(0.631084)*miR-4634+(0.52489)*miR-1238-5p+(-0.221116)*miR-3917-2.808	1.000	0.988	0.990	0.996
9	(0.522394)*miR-320a+(0.713656)*miR-665+(-0.668358)*miR-3184-5p+(-0.340122)*miR-6717-5p+(-1.09203)*miR-4459+(0.707769)*miR-6076+(0.461353)*miR-3195+(0.770345)*miR-1275+(0.252993)*miR-4454-9.880	1.000	0.994	0.995	0.997
9	(0.511236)*miR-320a+(0.707567)*miR-665+(-0.651788)*miR-3184-5p+(-0.350358)*miR-6717-5p+(-1.27837)*miR-4459+(0.807626)*miR-6076+(0.456295)*miR-3195+(0.760311)*miR-1275+(0.379116)*miR-1260b-9.516	1.000	0.994	0.995	0.997
9	(0.510011)*miR-320a+(0.815959)*miR-665+(-0.69174)*miR-3184-5p+(-0.35656)*miR-6717-5p+(-1.29477)*miR-4459+(0.788888)*miR-6076+(0.470717)*miR-3195+(0.76784)*miR-1275+(0.651645)*miR-5001-5p-11.951	1.000	0.993	0.994	0.997
9	(0.519745)*miR-320a+(0.531654)*miR-665+(-0.658821)*miR-3184-5p+(-0.324165)*miR-6717-5p+(-0.932908)*miR-4459+(0.33602)*miR-6076+(0.603606)*miR-3195+(0.631275)*miR-1275+(0.486329)*miR-1238-5p-8.260	1.000	0.994	0.994	0.997
9	(0.512828)*miR-320a+(0.76007)*miR-665+(-0.681578)*miR-3184-5p+(-0.307721)*miR-6717-5p+(-1.32506)*miR-4459+(0.767188)*miR-6076+(0.359621)*miR-3195+(0.813419)*miR-1275+(0.431744)*miR-940-8.563	1.000	0.994	0.994	0.997
9	(0.572027)*miR-320a+(0.585666)*miR-665+(-0.668993)*miR-3184-5p+(-0.303161)*miR-6717-5p+(-0.640325)*miR-6125+(0.624735)*miR-4634+(0.58289)*miR-1238-5p+(-0.303246)*miR-211-3p+(0.0598094)*miR-191-5p-1.854	0.994	0.993	0.993	0.997
9	(0.618001)*miR-320a+(0.455498)*miR-665+(-0.697685)*miR-3184-5p+(-0.25887)*miR-6717-5p+(-0.537925)*miR-6125+(0.676669)*miR-4634+(0.563153)*miR-1238-5p+(-0.391167)*miR-211-3p+(0.256619)*miR-658-3.722	0.994	0.994	0.994	0.997
9	(0.58907)*miR-320a+(0.499257)*miR-665+(-0.636291)*miR-3184-5p+(-0.293447)*miR-6717-5p+(-0.269283)*miR-6125+(0.520994)*miR-4634+(0.608465)*miR-1238-5p+(-0.839515)*miR-4749-5p+(0.224271)*miR-4690-5p-1.816	1.000	0.992	0.993	0.997
9	(0.563086)*miR-320a+(0.401015)*miR-665+(-0.594446)*miR-3184-5p+(-0.283484)*miR-6717-5p+(-0.825308)*miR-6125+(0.673469)*miR-4634+(0.526709)*miR-1238-5p+(-0.218113)*miR-614+(0.460326)*miR-1260b-2.889	1.000	0.992	0.993	0.997
9	(0.618112)*miR-320a+(0.759824)*miR-665+(-0.732543)*miR-3184-5p+(-0.280087)*miR-6717-5p+(-1.07416)*miR-4459+(0.619794)*miR-6076+(0.623858)*miR-3195+(0.678695)*miR-1275+(-0.190955)*miR-4516-5.594	1.000	0.990	0.991	0.997
10	(0.580879)*miR-320a+(0.690992)*miR-665+(-0.704358)*miR-3184-5p+(-0.312876)*miR-6717-5p+(-1.30221)*miR-4459+(0.728912)*miR-6076+(0.676143)*miR-3195+(0.716338)*miR-1275+(0.671553)*miR-3185+(-0.383997)*miR-4640-5p-9.375	1.000	0.995	0.996	0.997
10	(0.515217)*miR-320a+(0.688561)*miR-665+(-0.665552)*miR-3184-5p+(-0.344358)*miR-6717-5p+(-1.10995)*miR-4459+(0.709515)*miR-6076+(0.464601)*miR-3195+(0.781303)*miR-1275+(0.250435)*miR-4454+(0.0831167)*miR-4656-10.130	1.000	0.994	0.994	0.997

10	(0.530025)*miR-320a+(0.744335)*miR-665+(-0.679407)*miR-3184-5p+(-0.326181)*miR-6717-5p+(-1.08769)*miR-4459+(0.721842)*miR-6076+(0.45397)*miR-3195+(0.771364)*miR-1275+(0.240876)*miR-4454+(-0.153366)*miR-937-5p-8.860	1.000	0.994	0.995	0.997
10	(0.5017)*miR-320a+(0.722771)*miR-665+(-0.637715)*miR-3184-5p+(-0.353875)*miR-6717-5p+(-1.36653)*miR-4459+(0.826782)*miR-6076+(0.475137)*miR-3195+(0.759467)*miR-1275+(0.319278)*miR-1260b+(0.214079)*miR-4505-10.618	1.000	0.994	0.994	0.997
10	(0.500092)*miR-320a+(0.672231)*miR-665+(-0.647301)*miR-3184-5p+(-0.357022)*miR-6717-5p+(-1.30343)*miR-4459+(0.810879)*miR-6076+(0.459518)*miR-3195+(0.776402)*miR-1275+(0.378184)*miR-1260b+(0.117748)*miR-4656-9.990	1.000	0.994	0.995	0.997
10	(0.501818)*miR-320a+(0.748044)*miR-665+(-0.599732)*miR-3184-5p+(-0.334249)*miR-6717-5p+(-1.29359)*miR-4459+(0.864009)*miR-6076+(0.349839)*miR-3195+(0.735304)*miR-1275+(0.410398)*miR-1260b+(-0.497423)*miR-3620-5p-5.717	1.000	0.994	0.995	0.997
10	(0.478589)*miR-320a+(0.747746)*miR-665+(-0.650197)*miR-3184-5p+(-0.344806)*miR-6717-5p+(-1.38007)*miR-4459+(0.845208)*miR-6076+(0.346119)*miR-3195+(0.809699)*miR-1275+(0.249722)*miR-1260b+(0.299506)*miR-940-9.469	1.000	0.994	0.994	0.997
10	(0.512738)*miR-320a+(0.847023)*miR-665+(-0.689366)*miR-3184-5p+(-0.349493)*miR-6717-5p+(-1.21293)*miR-4459+(0.837817)*miR-6076+(0.443172)*miR-3195+(0.735491)*miR-1275+(0.787994)*miR-5001-5p+(-0.359121)*miR-4749-5p-	1.000	0.994	0.995	0.998
10	(0.536076)*miR-320a+(0.732235)*miR-665+(-0.682928)*miR-3184-5p+(-0.281853)*miR-6717-5p+(-1.29751)*miR-4459+(0.833301)*miR-6076+(0.333357)*miR-3195+(0.819766)*miR-1275+(0.463525)*miR-940+(-0.178021)*miR-365a-5p-8.301	1.000	0.994	0.995	0.997
10	(0.573741)*miR-320a+(0.699749)*miR-665+(-0.695261)*miR-3184-5p+(-0.336579)*miR-6717-5p+(-1.3866)*miR-4459+(0.837216)*miR-6076+(0.591009)*miR-3195+(0.548807)*miR-1275+(0.828173)*miR-4417+(0.52236)*miR-3185-15.645	1.000	0.989	0.990	0.998

Supplementary Table 4. Candidates of pivot miRNAs for model 2

	Cross-validated accuracy	AUC
miR-1233-5p	0.70	0.75
miR-4739	0.70	0.75
miR-4687-3p	0.69	0.75
miR-3679-3p	0.69	0.74
miR-4689	0.67	0.73
miR-557	0.67	0.68
miR-939-5p	0.67	0.72
miR-296-5p	0.66	0.71
miR-4271	0.66	0.72
miR-665	0.66	0.72

AUC; area uncer the receiver operating characteristic curve

Supplementary Table 5. Candidates of model 2

Number of miRNAs	Model candidates	Sensitivity	Specificity	Accuracy	AUC
1	(2.53937)*miR-4687-3p-23.8486	0.669	0.726	0.695	0.753
1	(2.14713)*miR-4271-17.0601	0.638	0.689	0.661	0.719
2	(2.10131)*miR-4687-3p+(1.2429)*miR-3663-3p-35.1832	0.794	0.748	0.773	0.820
2	(2.27871)*miR-4687-3p+(-1.27106)*miR-4741-9.6814	0.775	0.719	0.749	0.791
2	(2.09496)*miR-4687-3p+(-0.755759)*miR-939-5p-14.6983	0.763	0.763	0.763	0.836
2	(2.39361)*miR-4687-3p+(-1.11331)*miR-6089-7.4704	0.794	0.711	0.756	0.795
2	(2.46301)*miR-4687-3p+(1.09625)*miR-4758-5p-32.711	0.850	0.652	0.759	0.802
2	(2.18452)*miR-4687-3p+(-0.731224)*miR-4746-3p-15.339	0.838	0.682	0.766	0.804
2	(2.42236)*miR-4687-3p+(-0.839862)*miR-6125-12.3528	0.850	0.652	0.759	0.796
2	(2.15199)*miR-4687-3p+(0.548664)*miR-373-5p-23.9907	0.756	0.726	0.742	0.785
2	(2.5916)*miR-4687-3p+(-0.637309)*miR-4484-17.1862	0.788	0.711	0.753	0.779
2	(1.78977)*miR-4687-3p+(0.889947)*miR-3679-3p-22.8199	0.681	0.807	0.739	0.797
3	(1.1216)*miR-4687-3p+(-0.741221)*miR-939-5p+(1.73148)*miR-4739-25.0906	0.894	0.719	0.814	0.855
3	(1.65117)*miR-4687-3p+(-0.798603)*miR-939-5p+(0.776056)*miR-5739-15.353	0.925	0.689	0.817	0.871
3	(1.75669)*miR-4687-3p+(-0.814582)*miR-939-5p+(0.510742)*miR-4726-5p-14.4495	0.894	0.733	0.820	0.867
3	(0.905513)*miR-4271+(-0.757984)*miR-939-5p+(1.75513)*miR-4739-22.0538	0.856	0.733	0.800	0.856
3	(1.88999)*miR-4687-3p+(1.2135)*miR-3663-3p+(-0.700871)*miR-4746-3p-28.0707	0.831	0.741	0.790	0.859
3	(1.33952)*miR-4687-3p+(1.08352)*miR-3663-3p+(0.559124)*miR-1233-5p-32.8508	0.844	0.756	0.803	0.854
3	(1.30141)*miR-4271+(-0.750896)*miR-939-5p+(0.813109)*miR-3679-3p-10.6855	0.825	0.756	0.793	0.863
3	(1.80398)*miR-4687-3p+(1.43083)*miR-3663-3p+(-0.838266)*miR-187-5p-27.6427	0.856	0.733	0.800	0.858
3	(1.44525)*miR-4687-3p+(1.01046)*miR-3663-3p+(1.21199)*miR-4739-39.8120	0.813	0.763	0.790	0.832
3	(1.94337)*miR-4687-3p+(0.93569)*miR-3663-3p+(-0.624638)*miR-939-5p-25.7611	0.831	0.763	0.800	0.856
4	(1.75955)*miR-4687-3p+(-0.745539)*miR-939-5p+(0.675195)*miR-5739+(0.504613)*miR-4758-5p-20.6742	0.931	0.733	0.841	0.876
4	(1.60821)*miR-4687-3p+(-0.850013)*miR-939-5p+(0.61519)*miR-5739+(0.285639)*miR-4750-5p-15.4962	0.906	0.748	0.834	0.885
4	(1.17872)*miR-4687-3p+(-0.773777)*miR-939-5p+(1.47791)*miR-4739+(0.398193)*miR-4433a-5p-24.9766	0.875	0.763	0.824	0.873
4	(1.02019)*miR-4687-3p+(-0.829708)*miR-939-5p+(1.59877)*miR-4739+(0.395446)*miR-4750-5p-24.7262	0.881	0.748	0.820	0.880
4	(1.57602)*miR-4687-3p+(-0.766936)*miR-939-5p+(0.890386)*miR-5739+(-0.297136)*miR-211-3p-13.6029	0.913	0.748	0.837	0.879
4	(1.49581)*miR-4687-3p+(-0.837985)*miR-939-5p+(0.662009)*miR-5739+(0.353721)*miR-4726-5p-15.436	0.894	0.748	0.827	0.884
4	(1.7891)*miR-4687-3p+(-0.731397)*miR-939-5p+(0.726315)*miR-4726-5p+(-0.432796)*miR-3162-5p-13.6946	0.881	0.756	0.824	0.870
4	(1.16716)*miR-4687-3p+(1.34283)*miR-3663-3p+(-0.974174)*miR-187-5p+(0.695481)*miR-665-24.9827	0.781	0.874	0.824	0.895
4	(1.77952)*miR-4687-3p+(1.22158)*miR-3663-3p+(-0.976055)*miR-1915-3p+(0.344427)*miR-4433a-5p-23.423	0.881	0.756	0.824	0.865
4	(1.43175)*miR-4687-3p+(1.22258)*miR-3663-3p+(0.643598)*miR-665+(-0.604679)*miR-4707-3p-29.4933	0.850	0.800	0.827	0.858
5	(1.28428)*miR-4687-3p+(1.35037)*miR-3663-3p+(-0.805532)*miR-187-5p+(0.795039)*miR-665+(-0.865504)*miR-3197-19.1777	0.913	0.822	0.871	0.912

5	(0.664258)*miR-4687-3p+(-0.742472)*miR-939-5p+(1.86384)*miR-4739+(-1.09595)*miR-1469+(0.747502)*miR-1273g-3p-15.8132	0.894	0.830	0.864	0.926
5	(1.55307)*miR-4687-3p+(-0.664501)*miR-939-5p+(0.773433)*miR-5739+(0.389951)*miR-4726-5p+(-0.598922)*miR-1202-13.9803	0.919	0.763	0.848	0.888
5	(1.13626)*miR-4687-3p+(1.30033)*miR-3663-3p+(-0.808019)*miR-187-5p+(0.844329)*miR-665+(-0.588754)*miR-4745-5p-19.2526	0.844	0.867	0.854	0.914
5	(1.22129)*miR-4687-3p+(1.34421)*miR-3663-3p+(-0.837853)*miR-187-5p+(0.816053)*miR-665+(-0.644305)*miR-5787-18.5809	0.875	0.830	0.854	0.904
5	(1.70073)*miR-4687-3p+(-0.801332)*miR-939-5p+(0.559861)*miR-5739+(0.396639)*miR-4758-5p+(0.244012)*miR-4750-5p-19.6512	0.900	0.778	0.844	0.888
5	(1.55063)*miR-4687-3p+(-0.817373)*miR-939-5p+(0.609899)*miR-5739+(0.31203)*miR-4750-5p+(-0.103987)*miR-3622a-5p-14.7510	0.913	0.763	0.844	0.884
5	(1.64715)*miR-4687-3p+(-0.745306)*miR-939-5p+(0.642292)*miR-5739+(0.255522)*miR-4750-5p+(0.209082)*miR-4532-19.3107	0.925	0.756	0.848	0.890
5	(1.25045)*miR-4687-3p+(-0.750707)*miR-939-5p+(0.849945)*miR-5739+(-0.754436)*miR-211-3p+(0.700593)*miR-1273g-3p-12.7803	0.944	0.748	0.854	0.923
5	(1.20805)*miR-4687-3p+(1.34499)*miR-3663-3p+(-0.960688)*miR-187-5p+(0.727856)*miR-665+(-0.253523)*miR-3131-23.5149	0.888	0.793	0.844	0.896
6	(0.692339)*miR-4687-3p+(-0.725578)*miR-939-5p+(1.87934)*miR-4739+(-1.14196)*miR-1469+(0.710137)*miR-1273g-3p+(0.214351)*miR-486-3p-17.2526	0.906	0.844	0.878	0.927
6	(1.23654)*miR-4687-3p+(1.25149)*miR-3663-3p+(-0.769377)*miR-187-5p+(0.778652)*miR-665+(-0.940686)*miR-3197+(0.214004)*miR-373-5p-18.4301	0.906	0.844	0.878	0.918
6	(1.10929)*miR-4687-3p+(1.23101)*miR-3663-3p+(-0.738386)*miR-187-5p+(0.750154)*miR-665+(-0.940733)*miR-3197+(0.427597)*miR-3679-3p-18.3551	0.900	0.852	0.878	0.918
6	(1.21012)*miR-4687-3p+(-0.639705)*miR-939-5p+(0.864399)*miR-5739+(-0.868198)*miR-211-3p+(0.62848)*miR-1273g-3p+(0.85783)*miR-3663-3p-22.6265	0.931	0.837	0.888	0.944
6	(1.16211)*miR-4687-3p+(1.28692)*miR-3663-3p+(-0.599581)*miR-187-5p+(0.766891)*miR-665+(-1.31604)*miR-3197+(0.624088)*miR-5739-18.4154	0.938	0.844	0.895	0.929
6	(0.53768)*miR-4687-3p+(-0.702065)*miR-939-5p+(1.83738)*miR-4739+(-1.23536)*miR-1469+(0.721071)*miR-1273g-3p+(0.35529)*miR-373-5p-15.1885	0.913	0.844	0.881	0.933
6	(1.23215)*miR-4687-3p+(1.36217)*miR-3663-3p+(-0.804698)*miR-187-5p+(0.822725)*miR-665+(-0.756416)*miR-3197+(-0.140868)*miR-1469-18.4309	0.919	0.815	0.871	0.912
6	(1.27335)*miR-4687-3p+(1.26201)*miR-3663-3p+(-0.831124)*miR-187-5p+(0.794139)*miR-665+(-0.967339)*miR-3197+(0.226359)*miR-940-18.3047	0.913	0.822	0.871	0.914
6	(0.650546)*miR-4687-3p+(-0.711369)*miR-939-5p+(1.89137)*miR-4739+(-1.11533)*miR-1469+(0.706066)*miR-1273g-3p+(0.220264)*miR-4516-18.5186	0.906	0.859	0.885	0.927
6	(0.481504)*miR-4687-3p+(-0.740811)*miR-939-5p+(1.66813)*miR-4739+(-1.05294)*miR-1469+(0.697578)*miR-1273g-3p+(0.532284)*miR-3679-3p-15.4856	0.894	0.844	0.871	0.934
7	(1.14752)*miR-4687-3p+(-0.631265)*miR-939-5p+(0.822869)*miR-5739+(-0.850998)*miR-211-3p+(0.605939)*miR-1273g-3p+(0.883373)*miR-3663-3p+(0.072698)*miR-5008-5p-22.7678	0.881	0.896	0.888	0.945
7	(1.17945)*miR-4687-3p+(-0.654572)*miR-939-5p+(0.851088)*miR-5739+(-0.697005)*miR-211-3p+(0.711572)*miR-1273g-3p+(0.871141)*miR-3663-3p+(-0.358823)*miR-663a-20.0303	0.906	0.882	0.895	0.949

7	(1.18809)*miR-4687-3p+(-0.612487)*miR-939-5p+(0.812429)*miR-5739+(-0.844568)*miR-211-3p+(0.632017)*miR-1273g-3p+(0.915673)*miR-3663-3p+(-0.474416)*miR-3196-17.6291	0.894	0.904	0.898	0.949
7	(1.17576)*miR-4687-3p+(-0.621868)*miR-939-5p+(0.844428)*miR-5739+(-0.778765)*miR-211-3p+(0.648702)*miR-1273g-3p+(0.815076)*miR-3663-3p+(-0.608756)*miR-3656-15.6253	0.906	0.882	0.895	0.950
7	(1.18267)*miR-4687-3p+(-0.652565)*miR-939-5p+(0.831396)*miR-5739+(-0.667193)*miR-211-3p+(0.726054)*miR-1273g-3p+(0.850805)*miR-3663-3p+(-0.47873)*miR-1469-18.7	0.919	0.882	0.902	0.951
7	(1.05634)*miR-4687-3p+(-0.68144)*miR-939-5p+(0.785935)*miR-5739+(-0.935915)*miR-211-3p+(0.600218)*miR-1273g-3p+(0.882978)*miR-3663-3p+(0.379234)*miR-4726-5p-22.9233	0.850	0.948	0.895	0.954
7	(1.01227)*miR-4687-3p+(-0.590112)*miR-939-5p+(0.779474)*miR-5739+(-0.798329)*miR-211-3p+(0.680921)*miR-1273g-3p+(0.971261)*miR-3663-3p+(-0.975931)*miR-4508-10.3506	0.881	0.926	0.902	0.954
7	(1.15463)*miR-4687-3p+(1.35034)*miR-3663-3p+(-0.591174)*miR-187-5p+(0.699813)*miR-665+(-1.44186)*miR-3197+(0.609638)*miR-5739+(0.194139)*miR-423-5p-18.7904	0.919	0.867	0.895	0.932
7	(1.13047)*miR-4687-3p+(1.1763)*miR-3663-3p+(-0.577754)*miR-187-5p+(0.747301)*miR-665+(-1.38989)*miR-3197+(0.490015)*miR-5739+(0.148503)*miR-422a-16.1407	0.944	0.852	0.902	0.930
7	(1.05696)*miR-4687-3p+(1.30683)*miR-3663-3p+(-0.578755)*miR-187-5p+(0.766497)*miR-665+(-1.31955)*miR-3197+(0.622181)*miR-5739+(0.134303)*miR-3188-18.6638	0.944	0.852	0.902	0.930
8	(1.2657)*miR-4687-3p+(-0.674673)*miR-939-5p+(0.820031)*miR-5739+(-0.725625)*miR-211-3p+(0.684259)*miR-1273g-3p+(0.752968)*miR-3663-3p+(-0.442837)*miR-1469+(0.125934)*miR-4515-18.4984	0.931	0.889	0.912	0.952
8	(1.12291)*miR-4687-3p+(-0.612519)*miR-939-5p+(0.756662)*miR-5739+(-0.799246)*miR-211-3p+(0.67143)*miR-1273g-3p+(0.875382)*miR-3663-3p+(0.363729)*miR-4726-5p+(-0.425941)*miR-4745-5p-19.7051	0.925	0.889	0.909	0.958
8	(0.908288)*miR-4687-3p+(-0.666945)*miR-939-5p+(0.954738)*miR-5739+(-0.927838)*miR-211-3p+(0.654715)*miR-1273g-3p+(0.859535)*miR-3663-3p+(0.442768)*miR-4726-5p+(-0.317643)*miR-3917-21.1414	0.944	0.867	0.909	0.955
8	(0.923396)*miR-4687-3p+(-0.645126)*miR-939-5p+(0.820142)*miR-5739+(-0.917397)*miR-211-3p+(0.639747)*miR-1273g-3p+(0.809102)*miR-3663-3p+(0.460057)*miR-4726-5p+(-0.111395)*miR-6717-5p-21.23803	0.938	0.874	0.909	0.951
8	(1.11898)*miR-4687-3p+(-0.428363)*miR-939-5p+(0.860611)*miR-5739+(-0.692478)*miR-211-3p+(0.719494)*miR-1273g-3p+(0.999215)*miR-3663-3p+(-0.998211)*miR-4508+(-0.612028)*miR-1202-9.8436	0.913	0.911	0.912	0.954
8	(0.822601)*miR-4687-3p+(-0.619853)*miR-939-5p+(0.733417)*miR-5739+(-0.85622)*miR-211-3p+(0.633049)*miR-1273g-3p+(1.11161)*miR-3663-3p+(-1.17424)*miR-4508+(0.370776)*miR-423-5p-8.9759	0.900	0.926	0.912	0.956
8	(0.982418)*miR-4687-3p+(-0.643625)*miR-939-5p+(0.778015)*miR-5739+(-0.850388)*miR-211-3p+(0.647913)*miR-1273g-3p+(0.991446)*miR-3663-3p+(-1.13585)*miR-4508+(0.290292)*miR-4665-5p-9.6485	0.913	0.889	0.902	0.954

8	(0.800763)*miR-4687-3p+(-0.529073)*miR-939-5p+(0.93329)*miR-5739+(-0.701895)*miR-211-3p+(0.664847)*miR-1273g-3p+(0.916269)*miR-3663-3p+(-0.410787)*miR-614+(0.263676)*miR-4447-20.1840	0.925	0.874	0.902	0.955
8	(1.06363)*miR-4687-3p+(-0.482337)*miR-939-5p+(0.687298)*miR-5739+(-0.696628)*miR-211-3p+(0.759691)*miR-1273g-3p+(1.07911)*miR-3663-3p+(-0.138976)*miR-2467-3p+(-0.559442)*miR-187-5p-20.5538	0.925	0.919	0.922	0.952
8	(1.15906)*miR-4687-3p+(-0.609543)*miR-939-5p+(0.848362)*miR-5739+(-0.824904)*miR-211-3p+(0.607416)*miR-1273g-3p+(0.842225)*miR-3663-3p+(-0.574014)*miR-3196+(0.298917)*miR-762-19.3679	0.900	0.904	0.902	0.951
9	(0.980782)*miR-4687-3p+(-0.716971)*miR-939-5p+(0.670104)*miR-5739+(-0.797919)*miR-211-3p+(0.693442)*miR-1273g-3p+(0.977207)*miR-3663-3p+(0.395505)*miR-4726-5p+(-0.54103)*miR-4745-5p+(0.770664)*miR-1268b-	0.938	0.911	0.925	0.961
9	(1.08315)*miR-4687-3p+(-0.449295)*miR-939-5p+(0.67198)*miR-5739+(-0.683062)*miR-211-3p+(0.788234)*miR-1273g-3p+(1.09054)*miR-3663-3p+(-0.141813)*miR-2467-3p+(-0.499435)*miR-187-5p+(-0.420869)*miR-1237-5p-16.4354	0.931	0.904	0.919	0.954
9	(1.27981)*miR-4687-3p+(-0.662051)*miR-939-5p+(0.794449)*miR-5739+(-0.659323)*miR-211-3p+(0.766752)*miR-1273g-3p+(0.856939)*miR-3663-3p+(0.3655)*miR-4726-5p+(-0.361103)*miR-4745-5p+(-0.33779)*miR-642b-3p-	0.931	0.904	0.919	0.959
9	(0.925115)*miR-4687-3p+(-0.590973)*miR-939-5p+(0.785067)*miR-5739+(-0.812517)*miR-211-3p+(0.713659)*miR-1273g-3p+(0.755877)*miR-3663-3p+(0.422873)*miR-4726-5p+(-0.148111)*miR-6717-5p+(-0.621422)*miR-6089-	0.925	0.911	0.919	0.953
9	(0.989162)*miR-4687-3p+(-0.429491)*miR-939-5p+(0.8438)*miR-5739+(-0.677704)*miR-211-3p+(0.739417)*miR-1273g-3p+(1.01954)*miR-3663-3p+(-0.968201)*miR-4508+(-0.614224)*miR-1202+(0.171979)*miR-3188-10.5368	0.913	0.933	0.922	0.958
9	(1.05366)*miR-4687-3p+(-0.497262)*miR-939-5p+(0.679051)*miR-5739+(-0.701936)*miR-211-3p+(0.754126)*miR-1273g-3p+(1.07485)*miR-3663-3p+(-0.147024)*miR-2467-3p+(-0.523716)*miR-187-5p+(0.14121)*miR-937-5p-21.4704	0.931	0.911	0.922	0.953
9	(1.03366)*miR-4687-3p+(-0.47347)*miR-939-5p+(0.683634)*miR-5739+(-0.632083)*miR-211-3p+(0.775603)*miR-1273g-3p+(1.03677)*miR-3663-3p+(-0.145365)*miR-2467-3p+(-0.524357)*miR-187-5p+(-0.473578)*miR-3656-15.0152	0.931	0.904	0.919	0.953
9	(0.757008)*miR-4687-3p+(-0.607174)*miR-939-5p+(0.879044)*miR-5739+(-0.655401)*miR-211-3p+(0.695924)*miR-1273g-3p+(0.966629)*miR-3663-3p+(-0.263352)*miR-1469+(-0.343632)*miR-614+(0.338381)*miR-4726-5p-18.0892	0.944	0.889	0.919	0.963
9	(1.30532)*miR-4687-3p+(-0.760802)*miR-939-5p+(0.789955)*miR-5739+(-0.855968)*miR-211-3p+(0.668779)*miR-1273g-3p+(0.941753)*miR-3663-3p+(0.34377)*miR-4726-5p+(-0.724089)*miR-4442+(0.224718)*miR-423-5p-20.9893	0.931	0.904	0.919	0.955
9	(1.38697)*miR-4687-3p+(-0.747732)*miR-939-5p+(0.816128)*miR-5739+(-0.807154)*miR-211-3p+(0.677878)*miR-1273g-3p+(0.932771)*miR-3663-3p+(0.400989)*miR-4726-5p+(-0.602182)*miR-4442+(-0.255529)*miR-371a-5p-	0.925	0.911	0.919	0.957
10	(1.05066)*miR-4687-3p+(-0.7381)*miR-939-5p+(0.691109)*miR-5739+(-0.764549)*miR-211-3p+(0.697072)*miR-1273g-3p+(1.01538)*miR-3663-3p+(0.376972)*miR-4726-5p+(-0.56563)*miR-4745-5p+(0.773876)*miR-1268b+(-	0.931	0.926	0.929	0.963
10	(0.996007)*miR-4687-3p+(-0.740504)*miR-939-5p+(0.717848)*miR-5739+(-0.798134)*miR-211-3p+(0.719455)*miR-1273g-3p+(1.03608)*miR-3663-3p+(0.519928)*miR-4726-5p+(-0.582579)*miR-4745-5p+(0.785734)*miR-1268b+(-	0.938	0.919	0.929	0.965

10	(0.665763)*miR-4687-3p+(-0.525277)*miR-939-5p+(0.808861)*miR-5739+(-0.749447)*miR-211-3p+(0.607)*miR-1273g-3p+(0.993715)*miR-3663-3p+(-0.511094)*miR-614+(0.198392)*miR-4447+(0.45659)*miR-6722-3p+(1.06707)*miR-	0.938	0.919	0.929	0.962
10	(0.965991)*miR-4687-3p+(-0.713774)*miR-939-5p+(0.68686)*miR-5739+(-0.790011)*miR-211-3p+(0.699452)*miR-1273g-3p+(0.974254)*miR-3663-3p+(0.396523)*miR-4726-5p+(-0.543649)*miR-4745-5p+(0.75757)*miR-1268b+(-	0.938	0.904	0.922	0.961
10	(0.977491)*miR-4687-3p+(-0.715045)*miR-939-5p+(0.668288)*miR-5739+(-0.795276)*miR-211-3p+(0.693252)*miR-1273g-3p+(0.981269)*miR-3663-3p+(0.394344)*miR-4726-5p+(-0.537323)*miR-4745-5p+(0.783835)*miR-1268b+(-	0.931	0.919	0.925	0.961
10	(0.977347)*miR-4687-3p+(-0.717165)*miR-939-5p+(0.668333)*miR-5739+(-0.794453)*miR-211-3p+(0.694069)*miR-1273g-3p+(0.98269)*miR-3663-3p+(0.38735)*miR-4726-5p+(-0.528181)*miR-4745-5p+(0.786477)*miR-	0.938	0.911	0.925	0.961
10	(0.976032)*miR-4687-3p+(-0.718915)*miR-939-5p+(0.663642)*miR-5739+(-0.805682)*miR-211-3p+(0.68785)*miR-1273g-3p+(0.985944)*miR-3663-3p+(0.392992)*miR-4726-5p+(-0.539052)*miR-4745-5p+(0.775504)*miR-	0.944	0.911	0.929	0.961
10	(1.00775)*miR-4687-3p+(-0.732522)*miR-939-5p+(0.635253)*miR-5739+(-0.788043)*miR-211-3p+(0.675716)*miR-1273g-3p+(0.94963)*miR-3663-3p+(0.361252)*miR-4726-5p+(-0.515262)*miR-4745-5p+(0.756078)*miR-	0.944	0.911	0.929	0.959
10	(0.964278)*miR-4687-3p+(-0.704553)*miR-939-5p+(0.661099)*miR-5739+(-0.786611)*miR-211-3p+(0.700043)*miR-1273g-3p+(0.974343)*miR-3663-3p+(0.387908)*miR-4726-5p+(-0.525664)*miR-4745-5p+(0.849986)*miR-1268b+(-	0.931	0.919	0.925	0.961
10	(0.972866)*miR-4687-3p+(-0.711766)*miR-939-5p+(0.687063)*miR-5739+(-0.797589)*miR-211-3p+(0.693729)*miR-1273g-3p+(1.00288)*miR-3663-3p+(0.391204)*miR-4726-5p+(-0.532569)*miR-4745-5p+(0.771337)*miR-1268b+(-	0.931	0.919	0.925	0.960

Supplementary Table 6. Candidates of pivot miRNAs for model 3

	Cross-validated accuracy	AUC
miR-1273g-3p	0.65	0.70
miR-663b	0.65	0.68
miR-4294	0.63	0.61
miR-135a-3p	0.63	0.69
miR-550a-5p	0.63	0.61
miR-4787-3p	0.63	0.64
miR-658	0.62	0.68
miR-4463	0.62	0.63
miR-665	0.62	0.64
miR-4689	0.62	0.65

AUC; area uncer the receiver operating characteristic curve

Supplementary Table 7. Candidates of model 3

Number of miRNAs	Model candidates	Sensitivity	Specificity	Accuracy	AUC
1	(1.46003)*miR-663b-12.767	0.650	0.683	0.659	0.685
2	(0.981535)*miR-663b+(0.959865)*miR-658-14.992	0.681	0.667	0.677	0.718
2	(0.930841)*miR-663b+(-0.693426)*miR-4730-2.025	0.906	0.413	0.767	0.707
2	(1.26357)*miR-663b+(-1.66658)*miR-3940-5p+7.711	0.825	0.476	0.727	0.696
2	(1.06484)*miR-663b+(1.68856)*miR-4689-24.366	0.888	0.460	0.767	0.716
2	(1.24284)*miR-663b+(-1.17183)*miR-1915-3p+2.126	0.906	0.429	0.771	0.706
2	(1.27408)*miR-663b+(0.780393)*miR-5739-16.801	0.625	0.714	0.650	0.702
2	(1.55908)*miR-663b+(-1.34642)*miR-4787-5p+3.705	0.613	0.714	0.641	0.700
2	(1.25653)*miR-663b+(0.442675)*miR-4783-3p-14.355	0.794	0.476	0.704	0.675
2	(1.01111)*miR-663b+(-0.873745)*miR-4530-0.197	0.844	0.460	0.735	0.675
2	(1.07449)*miR-663b+(1.22772)*miR-6088-22.913	0.925	0.397	0.776	0.705
3	(0.89243)*miR-663b+(-0.659147)*miR-4730+(0.444242)*miR-1238-5p-5.066	0.981	0.349	0.803	0.695
3	(0.877983)*miR-663b+(-0.646502)*miR-4730+(0.51566)*miR-197-5p-5.933	0.994	0.349	0.812	0.708
3	(0.706476)*miR-663b+(-0.627041)*miR-4730+(0.488412)*miR-1233-5p-6.655	0.988	0.381	0.816	0.713
3	(0.883202)*miR-663b+(0.878992)*miR-658+(-1.07279)*miR-1915-3p-1.242	0.981	0.349	0.803	0.724
3	(0.866567)*miR-663b+(-0.686621)*miR-4730+(0.436775)*miR-4271-5.360	0.831	0.524	0.744	0.712
3	(0.88129)*miR-663b+(-0.63771)*miR-4730+(0.521412)*miR-4728-5p-5.673	0.988	0.349	0.807	0.710
3	(0.808618)*miR-663b+(-0.729348)*miR-4730+(0.582887)*miR-4726-5p-4.585	0.963	0.397	0.803	0.708
3	(1.1749)*miR-663b+(-1.24973)*miR-1915-3p+(0.493625)*miR-6132-0.765	0.969	0.381	0.803	0.692
3	(1.12266)*miR-663b+(-1.21045)*miR-1915-3p+(0.597026)*miR-744-5p-1.615	0.944	0.413	0.794	0.696
3	(1.28807)*miR-663b+(-1.67197)*miR-4787-5p+(1.15984)*miR-4707-5p+1.809	0.944	0.397	0.789	0.691
4	(0.719627)*miR-663b+(-0.722705)*miR-4730+(0.27386)*miR-642a-3p+(0.67719)*miR-658-6.250	0.994	0.397	0.825	0.721
4	(0.686092)*miR-663b+(-0.63574)*miR-4730+(0.270254)*miR-197-5p+(0.617069)*miR-658-6.348	0.994	0.397	0.825	0.719
4	(0.824659)*miR-663b+(-0.732745)*miR-4730+(0.447938)*miR-671-5p+(0.561016)*miR-658-7.547	0.950	0.460	0.812	0.746
4	(0.68059)*miR-663b+(-0.63997)*miR-4730+(0.243234)*miR-1238-5p+(0.652939)*miR-658-6.256	0.975	0.413	0.816	0.718
4	(0.805871)*miR-663b+(-0.584999)*miR-4730+(0.626978)*miR-197-5p+(-0.335177)*miR-1202-4.744	0.925	0.429	0.785	0.708
4	(0.64605)*miR-663b+(-0.633315)*miR-4730+(0.163123)*miR-4728-5p+(0.757818)*miR-658-6.927	0.731	0.651	0.709	0.728
4	(0.888667)*miR-663b+(-0.656539)*miR-4730+(0.463694)*miR-1238-5p+(-0.109386)*miR-498-4.657	0.963	0.365	0.794	0.697
4	(0.887595)*miR-663b+(-0.659298)*miR-4730+(0.435615)*miR-1238-5p+(0.0387368)*miR-4271-5.240	0.988	0.349	0.807	0.696
4	(1.00304)*miR-663b+(-0.548386)*miR-4730+(0.490218)*miR-1238-5p+(-0.274025)*miR-4745-5p-3.886	0.994	0.349	0.812	0.709
4	(0.923206)*miR-663b+(-0.635302)*miR-4730+(0.446231)*miR-1238-5p+(-0.0580659)*miR-204-3p-4.617	0.994	0.333	0.807	0.696
5	(0.627763)*miR-663b+(-0.748548)*miR-4730+(0.274339)*miR-642a-3p+(0.683873)*miR-658+(0.116157)*miR-486-3p-6.277	0.994	0.397	0.825	0.720

5	(0.731302)*miR-663b+(-0.716639)*miR-4730+(0.295067)*miR-642a-3p+(0.656081)*miR-658+(-0.0728855)*miR-486-5p-5.975	0.994	0.397	0.825	0.727
5	(0.660153)*miR-663b+(-0.758142)*miR-4730+(0.269332)*miR-642a-3p+(0.698012)*miR-658+(-0.16002)*miR-4433a-3p-4.317	0.994	0.397	0.825	0.721
5	(0.633188)*miR-663b+(-0.728701)*miR-4730+(0.220499)*miR-642a-3p+(0.662004)*miR-658+(0.20171)*miR-4656-6.511	0.994	0.397	0.825	0.723
5	(0.705212)*miR-663b+(-0.706338)*miR-4730+(0.214467)*miR-642a-3p+(0.625482)*miR-658+(0.345268)*miR-1207-5p-8.159	0.981	0.413	0.821	0.727
5	(0.79513)*miR-663b+(-0.654324)*miR-4730+(0.257877)*miR-642a-3p+(0.657262)*miR-658+(-0.195509)*miR-187-5p-5.685	0.994	0.397	0.825	0.715
5	(0.700302)*miR-663b+(-0.7378)*miR-4730+(0.277335)*miR-642a-3p+(0.690137)*miR-658+(-0.0921697)*miR-6716-5p-5.528	0.994	0.397	0.825	0.721
5	(0.733859)*miR-663b+(-0.718479)*miR-4730+(0.318864)*miR-642a-3p+(0.697373)*miR-658+(-0.117317)*miR-4513-6.149	0.994	0.397	0.825	0.725
5	(0.68965)*miR-663b+(-0.720139)*miR-4730+(0.261571)*miR-642a-3p+(0.69764)*miR-658+(-0.0757488)*miR-1225-5p-5.541	0.994	0.397	0.825	0.721
5	(0.703385)*miR-663b+(-0.626219)*miR-4730+(0.324514)*miR-197-5p+(0.590048)*miR-658+(-0.0728474)*miR-92a-3p-6.369	0.994	0.397	0.825	0.722
6	(0.625888)*miR-663b+(-0.753969)*miR-4730+(0.282244)*miR-642a-3p+(0.693964)*miR-658+(0.12838)*miR-486-3p+(-0.0106754)*miR-1246-6.375	0.994	0.397	0.825	0.720
6	(0.56693)*miR-663b+(-0.78447)*miR-4730+(0.269795)*miR-642a-3p+(0.704884)*miR-658+(0.117486)*miR-486-3p+(-0.160858)*miR-4433a-3p-4.330	0.994	0.397	0.825	0.717
6	(0.601032)*miR-663b+(-0.745652)*miR-4730+(0.280031)*miR-642a-3p+(0.709484)*miR-658+(0.125563)*miR-486-3p+(-0.099283)*miR-3185-5.567	0.994	0.397	0.825	0.725
6	(0.668213)*miR-663b+(-0.717353)*miR-4730+(0.216411)*miR-642a-3p+(0.62973)*miR-658+(0.0473248)*miR-486-3p+(0.335108)*miR-1207-5p-8.116	0.981	0.413	0.821	0.726
6	(0.649505)*miR-663b+(-0.742225)*miR-4730+(0.316582)*miR-642a-3p+(0.70223)*miR-658+(0.105576)*miR-486-3p+(-0.11023)*miR-4513-6.183	0.994	0.397	0.825	0.722
6	(0.591996)*miR-663b+(-0.771829)*miR-4730+(0.306822)*miR-642a-3p+(0.694057)*miR-658+(0.125283)*miR-486-3p+(0.263582)*miR-6724-5p-8.911	0.994	0.397	0.825	0.718
6	(0.627867)*miR-663b+(-0.744653)*miR-4730+(0.188691)*miR-642a-3p+(0.573779)*miR-658+(0.102049)*miR-486-3p+(0.204155)*miR-4726-5p-6.184	0.994	0.397	0.825	0.717
6	(0.634643)*miR-663b+(-0.73339)*miR-4730+(0.257948)*miR-642a-3p+(0.67272)*miR-658+(0.107833)*miR-486-3p+(0.0528361)*miR-4447-6.556	0.994	0.397	0.825	0.720
6	(0.639361)*miR-663b+(-0.763663)*miR-4730+(0.270659)*miR-642a-3p+(0.674911)*miR-658+(0.0980879)*miR-486-3p+(0.0349733)*miR-4792-6.247	0.994	0.397	0.825	0.719
6	(0.627268)*miR-663b+(-0.755394)*miR-4730+(0.266403)*miR-642a-3p+(0.681709)*miR-658+(0.11435)*miR-486-3p+(0.0264432)*miR-760-6.318	0.994	0.397	0.825	0.720
7	(0.567202)*miR-663b+(-0.787482)*miR-4730+(0.275729)*miR-642a-3p+(0.711733)*miR-658+(0.126435)*miR-486-3p+(-0.00784746)*miR-1246+(-0.156497)*miR-4433a-3p-4.428	0.994	0.397	0.825	0.719
7	(0.623428)*miR-663b+(-0.753084)*miR-4730+(0.275853)*miR-642a-3p+(0.695814)*miR-658+(0.127544)*miR-486-3p+(-0.0107955)*miR-1246+(0.0202881)*miR-4270-6.477	0.994	0.397	0.825	0.720
7	(0.650887)*miR-663b+(-0.705429)*miR-4730+(0.216857)*miR-642a-3p+(0.666247)*miR-658+(0.0697278)*miR-486-3p+(-0.0159234)*miR-1246+(0.367045)*miR-4763-3p-9.248	0.994	0.397	0.825	0.717
7	(0.667378)*miR-663b+(-0.725833)*miR-4730+(0.227891)*miR-642a-3p+(0.645779)*miR-658+(0.0665224)*miR-486-3p+(-0.0209847)*miR-1246+(0.358621)*miR-1207-5p-8.322	0.994	0.413	0.830	0.727

7	(0.647061)*miR-663b+(-0.745975)*miR-4730+(0.31903)*miR-642a-3p+(0.707442)*miR-658+(0.113816)*miR-486-3p+(-0.00665389)*miR-1246+(-0.103761)*miR-4513-6.245	0.994	0.397	0.825	0.723
7	(0.657837)*miR-663b+(-0.785403)*miR-4730+(0.31614)*miR-642a-3p+(0.696428)*miR-658+(0.107005)*miR-486-3p+(-0.0119269)*miR-1246+(-0.137812)*miR-371a-5p-5.479	0.994	0.397	0.825	0.722
7	(0.634155)*miR-663b+(-0.736167)*miR-4730+(0.263643)*miR-642a-3p+(0.682805)*miR-658+(0.121541)*miR-486-3p+(-0.0140668)*miR-1246+(0.0680552)*miR-4447-6.764	0.994	0.397	0.825	0.720
7	(0.638212)*miR-663b+(-0.771388)*miR-4730+(0.279598)*miR-642a-3p+(0.685936)*miR-658+(0.11079)*miR-486-3p+(-0.0125209)*miR-1246+(0.038135)*miR-4792-6.338	0.994	0.397	0.825	0.719
7	(0.625001)*miR-663b+(-0.76409)*miR-4730+(0.27201)*miR-642a-3p+(0.692011)*miR-658+(0.12715)*miR-486-3p+(-0.0118029)*miR-1246+(0.0368813)*miR-760-6.454	0.994	0.397	0.825	0.720
7	(0.62472)*miR-663b+(-0.76091)*miR-4730+(0.259139)*miR-642a-3p+(0.681739)*miR-658+(0.128687)*miR-486-3p+(-0.0103243)*miR-1246+(0.105501)*miR-4655-5p-6.848	0.994	0.397	0.825	0.721
8	(0.648522)*miR-663b+(-0.720726)*miR-4730+(0.132763)*miR-642a-3p+(0.58303)*miR-658+(0.0767023)*miR-486-3p+(-0.0417862)*miR-1246+(0.22669)*miR-1207-5p+(0.165949)*miR-4419b-6.914	0.994	0.413	0.830	0.727
8	(0.628879)*miR-663b+(-0.713071)*miR-4730+(0.0788639)*miR-642a-3p+(0.57951)*miR-658+(0.127237)*miR-486-3p+(-0.0583069)*miR-1246+(0.159219)*miR-4419b+(0.161736)*miR-1238-5p-6.335	0.994	0.429	0.834	0.717
8	(0.678449)*miR-663b+(-0.814839)*miR-4730+(0.139375)*miR-642a-3p+(0.569833)*miR-658+(-0.117091)*miR-486-3p+(-0.0469016)*miR-1246+(0.197952)*miR-4419b+(0.459395)*miR-3621-8.749	0.994	0.413	0.830	0.725
8	(0.571856)*miR-663b+(-0.787596)*miR-4730+(0.282997)*miR-642a-3p+(0.704851)*miR-658+(0.118669)*miR-486-3p+(-0.00480641)*miR-1246+(-0.158775)*miR-4433a-3p+(-0.0446681)*miR-498-4.149	0.994	0.397	0.825	0.720
8	(0.523853)*miR-663b+(-0.789551)*miR-4730+(0.271085)*miR-642a-3p+(0.715459)*miR-658+(0.129742)*miR-486-3p+(-0.00617803)*miR-1246+(-0.151266)*miR-4433a-3p+(0.0731915)*miR-4281-4.939	0.994	0.397	0.825	0.720
8	(0.5711)*miR-663b+(-0.78284)*miR-4730+(0.274945)*miR-642a-3p+(0.707238)*miR-658+(0.11941)*miR-486-3p+(-0.00920735)*miR-1246+(-0.161391)*miR-4433a-3p+(0.0129938)*miR-1307-3p-4.473	0.994	0.397	0.825	0.718
8	(0.561025)*miR-663b+(-0.788532)*miR-4730+(0.284758)*miR-642a-3p+(0.716354)*miR-658+(0.130325)*miR-486-3p+(-0.00691248)*miR-1246+(-0.160989)*miR-4433a-3p+(-0.0215769)*miR-4534-4.292	0.994	0.397	0.825	0.719
8	(0.562069)*miR-663b+(-0.791028)*miR-4730+(0.278875)*miR-642a-3p+(0.711069)*miR-658+(0.127682)*miR-486-3p+(-0.0039444)*miR-1246+(-0.167642)*miR-4433a-3p+(-0.0220587)*miR-614-4.117	0.994	0.397	0.825	0.720
8	(0.579665)*miR-663b+(-0.807356)*miR-4730+(0.293283)*miR-642a-3p+(0.708743)*miR-658+(0.118022)*miR-486-3p+(-0.0141458)*miR-1246+(-0.173983)*miR-4433a-3p+(-0.0571846)*miR-1185-1-3p-3.777	0.994	0.397	0.825	0.720
8	(0.567173)*miR-663b+(-0.787477)*miR-4730+(0.27571)*miR-642a-3p+(0.711728)*miR-658+(0.126438)*miR-486-3p+(-0.00785126)*miR-1246+(-0.156483)*miR-4433a-3p+(4.19221e-05)*miR-642b-3p-4.428	0.994	0.397	0.825	0.719
9	(0.652735)*miR-663b+(-0.720547)*miR-4730+(0.13895)*miR-642a-3p+(0.577848)*miR-658+(0.0708589)*miR-486-3p+(-0.0393609)*miR-1246+(0.226012)*miR-1207-5p+(0.165417)*miR-4419b+(-0.0346496)*miR-498-6.742	0.994	0.413	0.830	0.727

9	(0.62261)*miR-663b+(-0.720033)*miR-4730+(0.126057)*miR-642a-3p+(0.577202)*miR-658+(0.078459)*miR-486-3p+(-0.0405344)*miR-1246+(0.220327)*miR-1207-5p+(0.158921)*miR-4419b+(0.045654)*miR-211-3p-6.825	0.994	0.413	0.830	0.725
9	(0.648274)*miR-663b+(-0.71948)*miR-4730+(0.137141)*miR-642a-3p+(0.590278)*miR-658+(0.0712604)*miR-486-3p+(-0.0409567)*miR-1246+(0.232334)*miR-1207-5p+(0.167045)*miR-4419b+(-0.0164595)*miR-5196-5p-6.912	0.994	0.413	0.830	0.726
9	(0.645367)*miR-663b+(-0.738826)*miR-4730+(0.14017)*miR-642a-3p+(0.579146)*miR-658+(0.0764153)*miR-486-3p+(-0.0411462)*miR-1246+(0.227469)*miR-1207-5p+(0.164304)*miR-4419b+(0.0420129)*miR-1915-3p-7.212	0.994	0.413	0.830	0.726
9	(0.650835)*miR-663b+(-0.720501)*miR-4730+(0.133739)*miR-642a-3p+(0.582855)*miR-658+(0.0774548)*miR-486-3p+(-0.0417725)*miR-1246+(0.228145)*miR-1207-5p+(0.166833)*miR-4419b+(-0.00734645)*miR-4656-6.910	0.994	0.413	0.830	0.727
9	(0.664486)*miR-663b+(-0.727391)*miR-4730+(0.158659)*miR-642a-3p+(0.585782)*miR-658+(0.0654753)*miR-486-3p+(-0.0430193)*miR-1246+(0.233305)*miR-1207-5p+(0.168281)*miR-4419b+(-0.0579954)*miR-1229-5p-6.752	0.994	0.413	0.830	0.723
9	(0.647503)*miR-663b+(-0.719555)*miR-4730+(0.131497)*miR-642a-3p+(0.583251)*miR-658+(0.0769382)*miR-486-3p+(-0.0413628)*miR-1246+(0.22856)*miR-1207-5p+(0.165644)*miR-4419b+(0.0037354)*miR-1185-1-3p-6.953	0.994	0.413	0.830	0.727
9	(0.650227)*miR-663b+(-0.723799)*miR-4730+(0.139042)*miR-642a-3p+(0.582859)*miR-658+(0.0766346)*miR-486-3p+(-0.041973)*miR-1246+(0.229621)*miR-1207-5p+(0.169219)*miR-4419b+(-0.0203124)*miR-5739-6.855	0.994	0.413	0.830	0.727
9	(0.654467)*miR-663b+(-0.72101)*miR-4730+(0.135807)*miR-642a-3p+(0.583355)*miR-658+(0.076124)*miR-486-3p+(-0.041234)*miR-1246+(0.226883)*miR-1207-5p+(0.166369)*miR-4419b+(-0.00731687)*miR-642b-3p-6.924	0.994	0.413	0.830	0.727
9	(0.71536)*miR-663b+(-0.710129)*miR-4730+(0.254386)*miR-642a-3p+(0.62798)*miR-658+(0.013056)*miR-486-3p+(-0.0518918)*miR-1246+(0.316977)*miR-1207-5p+(0.179147)*miR-4419b+(-0.264376)*miR-6124-7.222	0.994	0.429	0.834	0.723
10	(0.652537)*miR-663b+(-0.719221)*miR-4730+(0.143695)*miR-642a-3p+(0.585463)*miR-658+(0.0649897)*miR-486-3p+(-0.0384424)*miR-1246+(0.231995)*miR-1207-5p+(0.166572)*miR-4419b+(-0.0351882)*miR-498+(-0.0174772)*miR-5196-5p-6.737	0.994	0.413	0.830	0.726
10	(0.649076)*miR-663b+(-0.743012)*miR-4730+(0.148529)*miR-642a-3p+(0.572707)*miR-658+(0.0701433)*miR-486-3p+(-0.038417)*miR-1246+(0.226938)*miR-1207-5p+(0.16334)*miR-4419b+(-0.0367795)*miR-498+(0.0521713)*miR-1915-3p-7.102	0.994	0.413	0.830	0.727
10	(0.653302)*miR-663b+(-0.720492)*miR-4730+(0.13918)*miR-642a-3p+(0.577815)*miR-658+(0.0710579)*miR-486-3p+(-0.0393624)*miR-1246+(0.226375)*miR-1207-5p+(0.165638)*miR-4419b+(-0.0345797)*miR-498+(-0.00182818)*miR-4656-6.741	0.994	0.413	0.830	0.727
10	(0.650407)*miR-663b+(-0.717742)*miR-4730+(0.136083)*miR-642a-3p+(0.578244)*miR-658+(0.0712715)*miR-486-3p+(-0.0382857)*miR-1246+(0.230466)*miR-1207-5p+(0.164674)*miR-4419b+(-0.0355463)*miR-498+(0.00893039)*miR-1185-1-3p-6.830	0.994	0.413	0.830	0.726
10	(0.653056)*miR-663b+(-0.721264)*miR-4730+(0.140298)*miR-642a-3p+(0.577901)*miR-658+(0.0709474)*miR-486-3p+(-0.0394475)*miR-1246+(0.226705)*miR-1207-5p+(0.166186)*miR-4419b+(-0.0340311)*miR-498+(-0.00471863)*miR-5739-6.732	0.994	0.413	0.830	0.727
10	(0.653599)*miR-663b+(-0.720589)*miR-4730+(0.139385)*miR-642a-3p+(0.577906)*miR-658+(0.0707853)*miR-486-3p+(-0.0392845)*miR-1246+(0.226042)*miR-1207-5p+(0.165479)*miR-4419b+(-0.0345826)*miR-498+(-0.0010741)*miR-642b-3p-6.744	0.994	0.413	0.830	0.727

10	(0.64995)*miR-663b+(-0.721115)*miR-4730+(0.139387)*miR-642a-3p+(0.577829)*miR-658+(0.0711051)*miR-486-3p+(-0.0395083)*miR-1246+(0.225649)*miR-1207-5p+(0.165064)*miR-4419b+(-0.0348617)*miR-498+(0.00689319)*miR-6090-6.802	0.994	0.413	0.830	0.726
10	(0.641043)*miR-663b+(-0.714209)*miR-4730+(0.129487)*miR-642a-3p+(0.551135)*miR-658+(0.0756538)*miR-486-3p+(-0.0400371)*miR-1246+(0.214518)*miR-1207-5p+(0.154943)*miR-4419b+(-0.0444118)*miR-498+(0.0970393)*miR-4690-5p-6.907	0.994	0.413	0.830	0.724
10	(0.650673)*miR-663b+(-0.723171)*miR-4730+(0.139603)*miR-642a-3p+(0.577692)*miR-658+(0.0725273)*miR-486-3p+(-0.0390928)*miR-1246+(0.226979)*miR-1207-5p+(0.164887)*miR-4419b+(-0.034611)*miR-498+(0.0156549)*miR-1237-5p-6.920	0.994	0.413	0.830	0.726
10	(0.613935)*miR-663b+(-0.721838)*miR-4730+(0.136304)*miR-642a-3p+(0.575945)*miR-658+(0.0765318)*miR-486-3p+(-0.038882)*miR-1246+(0.230897)*miR-1207-5p+(0.162166)*miR-4419b+(-0.0394251)*miR-498+(0.0623679)*miR-1469-7.092	0.994	0.413	0.830	0.725

Supplementary Table 8. Characteristics of each serum-miRNA cluster

Characteristics	CL1 (*N* = 104)			CL2 (*N* = 83)			CL3 (*N* = 116)			
	N	**Mean**	**SD**	***N***	**Mean**	**SD**	***N***	**Mean**	**SD**	***p***
Age, years		55.8	11.7		57.3	12.4		57.6	10.8	0.48[a]
Histopathological subtypes										0.22[b]
Serous	59			57			66			
Clear cell	23			17			24			
Endometrioid	14			8			21			
Mucinous	8			1			5			
Stage										0.65[b]
I	30			18			28			
II	10			6			13			
III – IV	64			59			75			

a; Student's t-test, b; χ^2 test

Supplementary Notes. Algorithm: combinatorial optimization for multicandidate miRNAs

Notation:

N: the number of candidate sets of miRNAs.

M: the maximum combination number of miRNAs in each candidate set.

Score: Accuracy = (TP + TN) / (TP+FP+FN+TN), where the meaning each variable is the following.

	cancer	no cancer
prediction positive	TP: number of true positives	FP: number of false positives
prediction negative	FN: number of false negatives	TN: number of true negatives

Step 1: Set N and M.

Step 2: FOR EACH miRNA:

Evaluate the average score (Accuracy) for a selected miRNA by means of linear discriminant analysis with leave one-out (LOO) cross validation.

Step 3: Sort the result in descending order, and maintain the top N miRNAs as the first candidate set to the next step.

Step 4: FOR i =2 TO M:

Step 4-1: FORE EACH N candidates:

Test all combinations of the remaining miRNAs against the selected candidate (set), and generate scores using linear discriminant analysis with LOO (same as step 2).

Step 4-2: Sort scores for whole combinations (N x (total number of miRNA - i)) and Select the top N sets of miRNA i-length combinations to the next step.
